# Gender Differences in Excessive Screen Time among Chinese High School Students in Henan Province

**DOI:** 10.3390/ijerph20010721

**Published:** 2022-12-30

**Authors:** Zhenti Cui, Ping Zou, Zihan Lin, Yingdong Cao, Yan Luo

**Affiliations:** 1 School of Nursing, Sias University, Zhengzhou 451100, China; 2 Health Science Center, Xi’an Jiaotong University, Xi’an 710061, China; 3 Scholar Practitioner Program, School of Nursing, Faculty of Education and Professional Studies, Nipissing University, Toronto, ON M5T 1V4, Canada

**Keywords:** excessive screen time, epidemiology, associated factors, gender difference, China

## Abstract

In a technology-driven society, adolescents are particularly vulnerable to the effects of excessive screen time, and gender disparities are notable. However, evidence on the gender difference in excessive screen time among Chinese high school students is scarce. This study examined gender differences in excessive screen time and its impact on mental health and urinary incontinence among 15,055 high school-aged adolescents, including 7514 (49.9%) males and 7541 (50.1%) females with the mean age of 16.72 (SD 0.88) in Henan Province, China. A stratified two-stage cluster sampling design was applied. Mental Health Inventory of Middle School Students and International Consultation on Incontinence Questionnaire—Short Form was used to collect data about mental health and urinary incontinence status. The percentage of students reporting excessive screen time was 9.4%, predominantly male (15.3% versus 3.5%; *p* < 0.001). Physical disease, living on campus, and cigarette or alcohol consumption were common factors associated with excessive screen time in both genders. Students in their second or third year showed significantly lower rates of excessive screen time than their peers. In males, younger age, active sexual behavior, chronic constipation, and having a mother with a Ph.D. degree were factors associated with a higher probability of reporting excessive screen time, while females living in a rural–urban continuum or whose mothers have a junior college degree were associated with higher odds of reporting excessive screen times. Excessive screen time was significantly positively correlated with mental health problems and urinary incontinence (*p* < 0.05). Results suggests the need to address excessive screen time and to focus separately on the mechanisms influencing excessive screen time in males and females.

## 1. Introduction

Screen time (ST) refers to time spent in front of a screen, including watching TV, playing computer games, using mobile devices, and so on [1]. The Canadian 24-Hour Movement Guidelines for Children and Youth, and the Children and Adolescents Digital Media Guidelines from the American Academy of Pediatrics recommend no more than two hours of daily recreational ST, beyond which is called excessive ST [2,3]. The percentage of children and adolescents with excessive ST ranges from 16.8% to 80.6%, and differs by region and ethnicity [4,5,6,7,8]. Evidence from a systematic review showed that the prevalence of excessive ST among Brazilian adolescents aged 10–19 years was 70.9%, predominantly female (66.3% versus 59.2% in males) [4]. The 2009–2010 Survey of Health Behavior in School-aged Children, a national representative sample of Canadian youths in grades 6–10, reported that 80.6% reflected excessive ST [9]. The most recent large-scale epidemiologic survey of excessive ST in mainland China was conducted in 2015–2016 among 7200 adolescents aged 13–18 years from six regions; an overall rate of 16.8% was reported [7]. In a school-based study of 23,543 children and adolescents aged 7–12 years in Zhejiang province, the rate of excessive ST was 42.4% [6]. However, not having an accurate portrayal of just high school students creates a challenge in obtaining an excessive ST picture.

There are a number of recognized risk factors for excessive social media use that can be generally categorized as follows: socio-demographic factors (i.e., younger age, lower school grade), poor physical condition, lifestyle (such as sexual activity, smoking, and alcohol consumption), and environmental factors (family, school, and social environment) [10,11,12,13]. Research has found that family environment—location, family structure, and parental education level—plays a mediating role in prospective ST by gender [11], and mothers’ behavior may have a greater impact on adolescents’ ST than the behavior of fathers [12]. For example, the frequency of mothers’ outdoor activities was significantly associated with outdoor activities in their children [12].

Evidence gathered over decades supports links between sedentary behavior, especially recreational ST, and poorer health outcomes among teenagers [14]. Previous studies have indicated that excessive ST has a strong correlation with chronic disease (e.g., cardiovascular diseases, diabetes, and obesity) and mental health problems; the indirect impact of excessive ST on mental health problems is mediated by lifestyle choices and social interactions [15]. In a systematic review of ST, the greatest gain in health was associated with shifting from inactivity to small amounts of physical activity and a reduction in ST [16]. Meanwhile, research has also shown that there is a link between sedentary behavior and urinary incontinence in females [17]. Adolescents with excessive ST might have delayed toileting behaviors, which is a risk factor of urinary incontinence [17]. We hypothesized that excessive ST negatively affects mental well-being and urinary incontinence because it displaces time participating in healthier activities, such as physical exercise, urinate regularly.

Although most studies have shown that gender is an independent factor associated with excessive ST [8], little research has been completed on gender differences in prevalence, associated factors, and the impact of ST among adolescents. Currently, most researchers concentrate on the health effects of various ST types on children and adolescents. Evidence indicates that excessive ST may cause more serious consequences for females than for males due to the physiological and psychological differences between gender [8]. Females were more likely to be introspective when exposed to social ST, which may increase the focus on self-objectification (greater emphasis on their physical appearance) [18]. As males and females are different in nature and have different reactions to the same factor, exploring separately the risk factors for males and females may be helpful in developing targeted interventions [19].

Henan province is in central China and is the most populous of the 31 mainland Chinese provinces and municipalities. According to 2018 data from the Ministry of Education of the People’s Republic of China, there were 23.67 million high school students in China, and more than 2 million resided in Henan province; it has more high school students than the other hinterland provinces, and students are currently under a high level of stress due to the very competitive National College Entrance Examination. The aim of the present study was to investigate the gender difference in prevalence, associated factors, and impact of excessive ST among high school students in Henan province, China.

## 2. Materials and Methods

### 2.1. Sampling and Setting

High school students were recruited based on a stratified two-stage cluster sampling from June to August 2018. Details of the design and sampling method of this study have been described previously [20]. Briefly, 46 schools in 17 cities around Henan Province were chosen for Stage 1 of the study based on probability proportionate to enrollment size. In Stage 2, two to three classes from each grade were randomly chosen in selected schools. Students were recruited using grades as strata. The head teachers of the chosen classes sent an invitation to all of the students to take part in the study.

### 2.2. Ethical Considerations

This study protocol has been approved by the Xi’an Jiaotong University Ethics Committee of the Health Science Center, as well as the local schools that participated (project identification code: 2018-296). Details of the study, data security, and consent were informed before participants filled out the questionnaires, and permission from all participants and their parents was gained.

### 2.3. Instruments

#### 2.3.1. Demographic Information

Participants’ demographic information sheet was collected in four parts, which included demographic factors [age, grade level, body mass index (BMI), ethnicity, and living on campus or not], health-related factors (physical disease and chronic constipation, as defined by the Rome IV criteria) [21], lifestyle (smoking, alcohol consumption, and sexual activity), and family factors (inhabitation, single parent, and parental education).

#### 2.3.2. High Screen Time

For this study, excessive ST was defined as “exceeding the recommended two hours of daily recreational ST”, in accordance with guidelines from the American Academy of Pediatrics and the Canadian 24-Hour Movement Guidelines for Children and Youth, and calculated by responses to the question: “Over the last 7 days, how many hours of recreational screen time did you spend on average per day, including watching TV, playing computer games, using mobile phones, and so on?” Students were given predefined categorical answers: less than half an hour (<0.5 h), half an hour to less than an hour (0.5–0.99 h), 1 h to 2 h (1–2 h), and more than 2 h (>2 h).

#### 2.3.3. Mental Health Inventory of Middle School Students (MMHI-60)

The MMHI-60, a self-administered screening test created to evaluate general mental health among Chinese middle and high school students was used to measure the mental health variable [22,23]. The MMHI-60 consists of 60 items reflecting 10 sub-scales: academic stress, emotional disturbance, obsessive–compulsive tendencies, anxiety, maladaptation, interpersonal sensitivity, paranoid ideation, depression, hostility, and psychological imbalance. Examples of the items include “Have you recently felt nervous and strung out?”, “Do you frequently think of committing suicide?”, and “Do you feel unable to solve problems?”, and participants should answer 1 (never), 2 (mild), 3 (moderate), 4 (serious), and 5 (very serious) for each item without reversed scoring. The total score of MMHI-60 and its sub-scale score were calculated by the average score of all items. A score of 2 was used as the cut-off point for mental health problems. The reliability and validity of the MMHI-60 have been well confirmed [20,23]. Test-retest reliability ranges from 0.716 to 0.873, and split-half reliability ranges from 0.634–0.873 [22]. The reported internal consistency was considered to be acceptable. In this study, the Cronbach’s alpha for the overall scale was 0.967 and varied amongst sub-scales from 0.684 to 0.862.

#### 2.3.4. International Consultation on Incontinence Questionnaire—Short Form (ICIQ-SF)

The urinary incontinence status of the participants was measured by the ICIQ-SF [24]. Questions about experiencing urinary leakage in the past four weeks included “unexplained and frequent incontinence before reaching a toilet, during sleep, after using a toilet”, meaning urgency urinary incontinence, or “incontinence triggered by coughing and/or sneezing, or physical activity”, meaning stress urinary incontinence. Participants who reported both types of symptoms were classified as having mixed urinary incontinence [25]. The ICIQ-SF score was calculated from three questions relating to frequency (0~5 points), severity (0~6 points), and condition-specific quality of life (0~10 points); a total score of 1 or above indicates urinary incontinence [26].

### 2.4. Statistical Analysis

All survey data were recorded using EpiData 3.1 software and double checked by two investigators. After obtaining and reviewing the data, R software (version 1.3.1093) was used for the statistical analysis. Continuous variables were expressed in mean ± standard deviation (SD), and categorical or ordinal data were expressed as absolute (*n*) and relative (%) frequencies. The percentage of participants with excessive ST (95% CI) was calculated for the overall population. In regression analysis, a stepwise logistic regression model was built for males and females separately with ST in a dichotomy as the dependent variable. After building the two models, the areas under the curve of each model were summarized. Then, the impact of ST on mental health problems and urinary incontinence was checked by conducting a chi-square test. Statistical significance was set at alpha = 0.05, and all tests are two-sided.

## 3. Results

### 3.1. Basic Characteristics of Participants

A total of 15,055 students were recruited for this survey, with a response rate of 91%—7514 (49.9%) males and 7541 (50.1%) females. Demographic characteristics of high school students are presented in Table 1. The mean age of the students was 16.72 (SD 0.88) years—16.78 (SD 0.88) years for males and 16.68 (SD 0.86) years for females (*p* < 0.001). More male participants than females were more likely to have physical disease (6.9% versus 5.9%, *p* = 0.011), smoke (7.9% versus 1.7%, *p* < 0.001), consume alcohol (19.8% versus 6.3%, *p* < 0.001), be sexually active (6.4% versus 2.5%, *p* < 0.001), and report mental health problems (43.3% versus 40.2%, *p* < 0.001). More female than male participants had parents with education levels of high school or below. Compared with their male counterparts, more females suffered chronic constipation (11.6% versus 9.5%, *p* < 0.001) and urinary incontinence (7.2% versus 6.0%, *p* < 0.001).

### 3.2. Rate of Excessive ST

Excessive ST overall was reported by 1410 of the 15,055 participants—9.4% (95% CI 8.9–9.8)—predominantly in males (15.3% versus 3.5% in females, *p* < 0.001). For both males and females, the percentage of those with excessive ST decreased with age from 10.6% at 14–16 years old to 8.6% at 17–20 years old (*p* < 0.05). Individuals whose mothers’ education level was Ph.D. reported the highest rate, with a significantly higher prevalence in males than in females (40.3% versus 21.0%, *p* < 0.001). Detailed information is shown in Table 2.

### 3.3. Factors Associated with Excessive ST

A univariate analysis of demographic data, physical disease, lifestyle, and familial factors associated with excessive ST is presented in Table 2. For both gender, younger age, studying in first-year, cigarettes, or alcohol consumption, having physical disease or chronic constipation, engaging in sexual behavior, living in a rural–urban continuum, and having a father or mother with a Ph.D. degree were associated with higher risk of excessive ST (*p* < 0.05). For males, living on campus was associated with higher odds of having excessive ST (*p* < 0.05). For females, having a single-parent family was found to be associated with higher odds of excessive ST (*p* < 0.05). Normal BMI was related to lower odds of reporting excessive ST (*p* < 0.05). The strongest associated factor for excessive ST was having a mother with a Ph.D. degree, for both males (OR = 3.654, 95% CI 2.567–5.167) and females (OR = 8.155, 95% CI 4.179–14.855).

Multivariate analysis results of this study are presented in Table 3. Multivariate analysis found that residing on campus, cigarettes or alcohol consumption, and physical disease were common factors associated with higher odds for excessive ST in both males and females (*p* < 0.05). Excessive ST was less likely to be reported among students in the second or third year (*p* < 0.05). The odds of excessive ST were greater in males engaging in sexual behavior (OR = 1.768, 95% CI 1.396–2.228), or having a mother with a Ph.D. degree (OR = 2.628, 95% CI 1.234–5.671), whereas the odds were greater among females living in a rural–urban continuum (OR = 2.361, 95% CI 1.430–3.768), or with a mother having a junior college degree (OR = 1.674, 95% CI 1.024–2.673). Older males were less likely to reporting excessive ST (OR = 0.88, 95% CI 0.807–0.96) or whose mother had a junior college degree (OR = 0.661, 95% CI 0.50–0.867) or a bachelor’s degree (OR = 0.521, 95% CI 0.375–0.716). For females, having a father with a junior college degree was relevant to lower odds of having excessive ST (OR = 0.450, 95% CI 0.260–0.749). The overall models were statistically significant for both genders, with the overall percentage for correction prediction at 69.0% in all participants, 67.8% in males and 70% in females, respectively (*p* < 0.001).

### 3.4. Impact of Excessive ST on Mental Health Problems and Urinary Incontinence

The impact of excessive ST on mental health problems and urinary incontinence is shown in Table 4. For both males and females, there is a significant difference in the proportion of mental health problems and its 10 dimensions between excessive ST and low ST (*p* < 0.001). Excessive ST is associated with a higher prevalence of urinary incontinence, and this difference is reflected mainly in urgency urinary incontinence (*p* < 0.05).

## 4. Discussion

This study produced several important findings. First, the findings indicate that 9.4% of Chinese adolescents aged 14–20 years report excessive ST, and the rate is higher in males (15.3% versus 3.5% in females). Second, greater odds of having excessive ST were strongly associated with residing on campus, cigarettes or alcohol consumption, and physical disease in both males and females. Finally, excessive ST was significantly associated with a higher prevalence of mental health problems and urinary incontinence, especially urgency urinary incontinence.

The present study revealed a 9.4% prevalence of excessive ST, which is lower than the wide range of excessive ST occurrence (16.8–80.6%) previously reported [4,5,6,7,8]. The relatively high learning intensity, school regulation, and fear of myopia may contribute to the lower percentages identified in this study. In addition, Henan Province is the most populous of the 31 provinces in Mainland China, which leads to fierce competition on college entrance examinations and the prohibition of high school students’ use of mobile phones or other electronic devices in classes [20]. Moreover, the incidence of myopia in Chinese children and adolescents ranks first in the world, which increases the focus of parents and high school students on protecting their vision, resulting in a reduction in ST [27].

In our study, males were 4.55 times more likely than females to have excessive ST. Previous research on gender differences in excessive ST has been inconsistent, with more studies indicating higher rates in men [6,28,29]. However, a systematic review of evidence among Brazilian adolescents claimed that there was no difference between genders [4]. One possible reason for gender differences might be the excessive use of video/computer games among males and more leisure reading among females [28]. Historically, when a summation of time spent in screen-specific behaviors (i.e., television + video/computer games) was applied, boys were found to report more screen time than girls [6,28,29]. There is a need for tailored treatment and intervention techniques since these sex-specific risk variables may differentially predispose male and female adolescents to excessive ST.

Younger age was associated with higher odds of excessive ST in males than females in our study, which aligns with previous studies finding that gender disparities in lifestyle change with increasing age [30]. With age, male students may engage in more physical activity to relieve academic stress than using electronic devices, while females may be more likely to adopt sedentary behavior, especially recreational ST [30]. Both male and female adolescents in their second or third year of study had lower odds of exhibiting excessive ST than students in the first year. One possible explanation may be that second- or third-year students need to pay more attention to their studies because they are nearing their entrance to university.

The probabilities of excessive ST were substantially associated with physical illness in both men and women, which validates earlier research demonstrating that excessive and addictive use of digital media correlates with adverse physical (e.g., obesity, metabolic syndrome, diabetes, cardiovascular disease, and impaired vision), psychological, social, and neurological conditions [14]. Studies have suggested that excessive ST is associated with poor sleep, unfavorable body composition, and higher clustered cardiometabolic risk scores, such as high blood pressure, obesity, low HDL cholesterol, poor stress regulation (high sympathetic arousal and cortisol dysregulation), and insulin resistance. Chronic constipation was significantly associated with excessive ST in males rather than females in our study. This supports the results of the earlier study, which showed a strong relationship between chronic constipation and high ST [31]. As fewer studies investigated the impact of chronic constipation on the risk of excessive ST with gender differences, there was no strong evidence to explain the gender disparity. A possible explanation for this gender difference might be that males with chronic constipation reported more loss of work productivity and activity impairment than females [31].

Unhealthy behaviors, including smoking and alcohol consumption, were significantly linked with excessive ST in both males and females in our study, reinforcing the findings of previous studies [32,33]. According to Jessop’s conceptual framework, unhealthy behaviors occur in clusters of multiple risk behaviors, rather than in isolation [34]. A two-year cohort study of Canadian adolescents found that smoking was likely to co-occur and interact with the excessive use of screens [32]. Observational learning, a key construct in Bandura’s social cognitive theory, may explain how ST media can affect adolescents’ risk-taking behavior in the context of Jessop’s framework [35]. Their behavior may be impacted by their exposure to violent, sexual, drug, or alcohol content on screens, especially given the volume of exposure, since adolescents may pick up behaviors by watching others do them, which increases their interest in them [33]. Given the clustering and co-occurring of unhealthy behavioral patterns, intervention strategies to promote healthy lifestyles should take a holistic approach by targeting multiple behavioral changes simultaneously.

Our findings are in line with previous studies indicating the significant interactions of high adolescent ST and familial factors. In this study, residing on campus, coming from a household that lives somewhere along the rural-urban continuum, and parental education level were all familial characteristics linked to excessive ST among high school students. This is consistent with a Chinese study that found that ST levels are higher among adolescents and children living on campus [36]. One possible explanation is that students living at home may be subject to more parental supervision than students living on campus [36]. Our study consistently reported inequality between adolescent ST and the degree of parental education [37,38]. Compared to sons with mothers had junior college or bachelor’s degrees, males of mothers with less education (a maximum of lower-secondary level) were more likely to present a higher ST. The findings are largely consistent with previous research and serve to highlight population groups that may be suitable for targeted intervention programs [37,38]. However, inconsistent association of maternal educational levels and male adolescents’ ST was found in this study, in which the sons of mothers with Ph.D. degrees were more likely to register excessive ST than their peers of mothers with lower levels of education (a maximum of the lower-secondary level). The proxy mechanisms between parental education and students’ ST remains unclear, and further prospective cohort studies are warranted to validate.

Excessive ST is strongly associated with mental health problems in our study, which is in line with previous studies [14]. Excessive ST may reduce social interaction and engagement, thus increasing the risk of mental health problems, such as feelings of loneliness, the likelihood of depression, and anxiety [39]. Apart from the psychological impact of excessive ST, its physiological impact should not be ignored. In our study, excessive ST was significantly associated with urinary incontinence, especially urgency urinary incontinence. Our results are in line with previous research investigating the determinants of urinary incontinence that concluded that prolonged sedentary behavior is associated with the emergence of urinary incontinence among adolescents [40].

The large population-based sample and the stratified cluster sampling design are two aspects of this study’s strengths. However, this study had several limitations. Firstly, the use of self-reported information on screen time, mental health problems, and experiences with incontinence might increase response bias. The responses might have been skewed in favor of socially acceptable facts, which could have led to overestimations or underestimations of the genuine relationships. Secondly, the nature of cross-sectional study design of our study prevents determinations of the causal relationships between excessive ST and associated factors. Finally, all the students were recruited from Henan province in China, which decreased the reliability of results generalizing to the entire high school students in China.

## 5. Conclusions

In the current study, it was discovered that the associated factors for excessive ST varied across males and females, providing support for customized therapies aimed at various high-risk populations. The study also provides novel evidence about the impact on mental health problems and urinary incontinence of excessive ST for males and females separately. These findings are helpful for enhancing the awareness of the impact of excessive ST and developing future targeted interventions on excessive ST. Future research on the effectiveness of particular therapies addressing modifiable risk factors will undoubtedly require more longitudinal cohort studies and randomized controlled trials.

## Figures and Tables

**Table 1:** Major characteristics of the study population (*n* = 15,055).

Variables		Total	Males	Females	*p* Value
Total		15,055	7514 (49.9)	7541 (50.1)	
Age	14–16	5881 (39.1)	2766 (36.8)	3115 (41.3)	<0.001 *
	17–20	9174 (60.9)	4748 (63.2)	4426 (58.7)	
BMI	<18.5	1346 (9.1)	899 (12.2)	447 (6.0)	<0.001
	18.5–24	12,294 (82.7)	5626 (76.4)	6668 (88.9)	
	>24	1226 (8.2)	843 (11.4)	383 (5.1)	
Grade	First year	5279 (35.1)	2678 (35.6)	2601 (34.5)	<0.001
	Second year	4009 (26.6)	2083 (27.7)	1926 (25.5)	
	Third year	5620 (37.3)	2672 (35.6)	2948 (39.1)	
	Fourth year ^§^	147 (1.0)	81 (1.1)	66 (0.9)	
Ethnicity	Han	14,679 (97.5)	7321 (97.4)	7358 (97.6)	0.577
	Minority	376 (2.5)	193 (2.6)	183 (2.4)	
Residence on campus	No	4047 (26.9)	2081 (27.7)	1966 (26.1)	0.025
	Yes	11,008 (73.1)	5433 (72.3)	5575 (73.9)	
Physical disease	No	14,086 (93.6)	6992 (93.1)	7094 (94.1)	0.011
	Yes	969 (6.4)	522 (6.9)	447 (5.9)	
Chronic constipation	No	13,471 (89.5)	6802 (90.5)	6669 (88.4)	<0.001
	Yes	1584 (10.5)	712 (9.5)	872 (11.6)	
Smoking	No	14,333 (95.2)	6921 (92.1)	7412 (98.3)	<0.001
	Yes	722 (4.8)	593 (7.9)	129 (1.7)	
Alcohol consumption	No	13,091 (87.0)	6025 (80.2)	7066 (93.7)	<0.001
	Yes	1964 (13.0)	1489 (19.8)	475 (6.3)	
Sexual activity	Never active	14,381 (95.5)	7030 (93.6)	7351 (97.5)	<0.001
	Ever active	674 (4.5)	484 (6.4)	190 (2.5)	
Inhabitation	Urban	5984 (39.7)	3096 (41.2)	2888 (38.3)	<0.001
	Rural	8411 (55.9)	4066 (54.1)	4345 (57.6)	
	Rural–urban continuum	660 (4.4)	352 (4.7)	308 (4.1)	
Single parent	No	14,061 (93.4)	7049 (93.8)	7012 (93.0)	0.041
	Yes	994 (6.6)	465 (6.2)	529 (7.0)	
Paternal education	High school and below	10,790 (71.7)	5308 (70.6)	5482 (72.7)	<0.001
	Junior college	2208 (14.7)	1093 (14.5)	1115 (14.8)	
	Bachelor	1700 (11.3)	877 (11.7)	823 (10.9)	
	Master	140 (0.9)	88 (1.2)	52 (0.7)	
	Ph.D.	217 (1.4)	148 (2.0)	69 (0.9)	
Maternal education	High school and below	11,436 (76.0)	5628 (74.9)	5808 (77.0)	<0.001
	Junior college	1748 (11.6)	885 (11.8)	863 (11.4)	
	Bachelor	1569 (10.4)	805 (10.7)	764 (10.1)	
	Master	101 (0.7)	57 (0.8)	44 (0.6)	
	Ph.D.	201 (1.3)	139 (1.8)	62 (0.8)	
Excessive screen time	No	13,645 (90.6)	6366 (84.7)	7279 (96.5)	<0.001
	Yes	1410 (9.4)	1148 (15.3)	262 (3.5)	
Mental health problems	No	8768 (58.2)	4259 (56.7)	4509 (59.8)	<0.001
	Yes	6287 (41.8)	3255 (43.3)	3032 (40.2)	
Urinary incontinence	No	14,060 (93.4)	7060 (94.0)	7000 (92.8)	<0.001
	Yes	995 (6.6)	454 (6.0)	541 (7.2)	

Abbreviations: BMI, body mass index. ^§^ students need to retake the National College Entrance Examination, and they take another year of high school with third-year students. * Calculated from Chi-square test, indicating the association between gender the variables in the first column.

**Table 2:** Univariate analysis of factors associated with excessive screen time.

Variables		All Participants		Males		Females	
		Positive Rate (95% CI)	OR (95% CI)	Positive Rate (95% CI)	OR (95% CI)	Positive Rate (95% CI)	OR (95% CI)
Total		9.4 (8.9, 9.8)	-	15.3 (14.5, 16.1)	-	3.5 (3.1, 3.9)	-
Gender	Female	3.5 (3.1, 3.9)	Ref.	-	-	-	-
	Male	15.3 (14.5, 16.1)	5.051 (4.386, 5.814)	-	-	-	-
Age	14–16	10.6 (9.8, 11.4)	Ref.	17.9 (16.5, 19.4)	Ref.	4.0 (3.4, 4.8)	Ref.
	17–20	8.6 (8.0, 9.2)	0.797 (0.714, 0.890)	13.8 (12.8, 14.8)	0.732 (0.644, 0.831)	3.1 (2.6, 3.6)	0.752 (0.588, 0.963)
BMI	<18.5	12.8 (11.2, 14.6)	Ref.	16.6 (14.4, 19.0)	Ref.	4.7 (3.1, 7.1)	Ref.
	18.5–24	8.3 (7.8, 8.8)	0.537 (0.457, 0.634)	14.1 (13.3, 15.1)	0.712 (0.595, 0.856)	3.2 (2.8, 3.7)	0.614 (0.404, 0.980)
	>24	10.5 (9.0, 12.3)	0.812 (0.645, 1.020)	13.1 (11.1, 15.5)	0.765 (0.595, 0.982)	4.5 (2.8, 7.0)	0.962 (0.511, 1.793)
Grade	First year	12.7 (11.9, 13.7)	Ref.	20.2 (18.7, 21.8)	Ref.	5.0 (4.3, 6.0)	Ref.
	Second year	7.6 (6.8, 8.4)	0.561 (0.486, 0.646)	11.8 (10.5, 13.3)	0.529 (0.449, 0.622)	3.0 (2.39, 3.8)	0.575 (0.416, 0.785)
	Third year	7.4 (6.7, 8.1)	0.547 (0.480, 0.621)	12.9 (11.7, 14.2)	0.584 (0.503, 0.676)	2.4 (1.9, 3.0)	0.465 (0.345, 0.622)
	Fourth year	13.6 (9.0, 20.1)	1.080 (0.650, 1.701)	21.0 (13.5, 31.1)	1.049 (0.591, 1.764)	4.6 (1.6, 12.5)	0.898 (0.217, 2.461)
Ethnicity	Han	9.3 (8.9, 9.8)	Ref.	15.3 (14.5, 16.1)	Ref.	3.4 (3.0, 3.9)	Ref.
	Minority	10.4 (7.7, 13.9)	1.123 (0.791, 1.551)	15.0 (10.7, 20.8)	0.980 (0.644, 1.438)	5.5 (3.0, 9.8)	1.630 (0.797, 2.964)
Residence on campus	No	6.6 (5.9, 7.4)	Ref.	10.2 (9.0, 11.6)	Ref.	2.8 (2.2, 3.6)	Ref.
	Yes	10.4 (9.8, 11.0)	1.632 (1.424, 1.877)	17.2 (16.2, 18.2)	1.823 (1.559, 2.140)	3.7 (3.3, 4.2)	1.340 (0.998, 1.828)
Physical disease	No	9.0 (8.5, 9.4)	Ref.	14.8 (14.0, 15.6)	Ref.	3.2 (2.8, 3.7)	Ref.
	Yes	15.3 (13.2, 17.7)	1.832 (1.518, 2.196)	22.0 (18.7, 25.8)	1.630 (1.307, 2.019)	7.4 (5.3, 10.2)	2.390 (1.610, 3.439)
Chronic constipation	No	8.9 (8.4, 9.4)	Ref.	14.4 (13.6, 15.3)	Ref.	3.3 (2.9, 3.7)	Ref.
	Yes	13.4 (11.8, 15.2)	1.583 (1.351, 1.847)	23.7 (20.8, 27.0)	1.851 (1.534, 2.224)	4.9 (3.7, 6.6)	1.528 (1.080, 2.113)
Smoking	No	8.5 (8.1,9.0)	Ref.	14.2 (13.4, 15.0)	Ref.	3.2 (2.9, 3.7)	Ref.
	Yes	26.2 (23.1, 29.5)	3.808 (3.187, 4.532)	28.0 (24.5, 31.7)	2.351 (1.937, 2.842)	17.8 (12.2, 25.3)	6.512 (3.985, 10.223)
Alcohol consumption	No	7.6 (7.2, 8.1)	Ref.	13.1 (12.3, 14.0)	Ref.	3.0 (2.6, 3.4)	Ref.
	Yes	20.9 (19.2, 22.8)	3.203 (2.821, 3.633)	24.1 (22.0, 26.4)	2.108 (1.831, 2.424)	11.0 (8.5, 14.1)	4.013 (2.891, 5.480)
Sexual activity	Never active	8.7 (8.3, 9.2)	Ref.	14.4 (13.6, 15.3)	Ref.	3.3 (2.9, 3.7)	Ref.
	Ever active	22.9 (19.8, 26.2)	3.095 (2.555, 3.729)	27.7 (23.9, 31.8)	2.271 (1.836, 2.795)	10.5 (6.9, 15.7)	3.456 (2.076, 5.458)
Inhabitation	Urban	8.3 (7.6, 9.0)	Ref.	12.8 (11.6, 14.0)	Ref.	3.5 (2.9, 4.2)	Ref.
	Rural	9.8 (9.2, 10.5)	1.209 (1.077, 1.360)	17.0 (15.9, 18.2)	1.398 (1.223, 1.598)	3.2 (2.7, 3.7)	0.908 (0.699, 1.182)
	Rural–urban continuum	13.3 (11.0, 16.1)	1.706 (1.331, 2.164)	17.9 (14.3, 22.2)	1.491 (1.105, 1.984)	8.1 (5.6, 11.7)	2.463 (1.532, 3.821)
Single parent	No	9.3 (8.9, 9.8)	Ref.	15.3 (14.4, 16.1)	Ref.	3.4 (3.0, 3.8)	Ref.
	Yes	10.1 (8.3, 12.1)	1.089 (0.874, 1.342)	15.7 (12.7, 19.3)	1.035 (0.794, 1.331)	5.1 (3.5, 7.3)	1.551 (1.009, 2.291)
Paternal education	High school and below	9.7 (9.2, 10.3)	Ref.	16.3 (15.3, 17.3)	Ref.	3.4 (3.0, 3.9)	Ref.
	Junior college	6.0 (5.1, 7.1)	0.590 (0.488, 0.709)	10.1 (8.4, 12.0)	0.576 (0.465, 0.708)	2.0 (1.3, 3.0)	0.573 (0.357, 0.875)
	Bachelor	8.6 (7.4, 10.0)	0.872 (0.725, 1.042)	12.5 (10.5, 14.9)	0.739 (0.594, 0.910)	4.4 (3.2, 6.0)	1.302 (0.891, 1.852)
	Master	9.3 (5.5, 15.2)	0.951 (0.510, 1.623)	11.4 (6.3, 19.7)	0.660 (0.320, 1.220)	5.8 (2.0 15.6)	1.743 (0.421, 4.803)
	Ph.D.	32.3 (26.4, 38.7)	4.422 (3.287, 5.895)	37.2 (29.8, 45.2)	3.046 (2.154, 4.269)	21.7 (13.6, 32.8)	7.909 (4.239, 13.924)
Maternal education	High school and below	9.6 (9.1, 10.2)	Ref.	16.2 (15.3, 17.2)	Ref.	3.2 (2.7, 3.6)	Ref.
	Junior college	7.0 (5.9, 8.3)	0.707 (0.580, 0.855)	10.3 (8.5, 12.5)	0.591 (0.468, 0.739)	3.6 (2.5, 5.1)	1.145 (0.763, 1.662)
	Bachelor	9.2 (7.4, 11.4)	0.690 (0.558, 0.843)	9.2 (7.4, 11.4)	0.522 (0.404, 0.665)	4.3 (3.1, 6.0)	1.388 (0.935, 1.998)
	Master	14.9 (9.2, 23.1)	1.644 (0.910, 2.767)	22.8 (13.8, 35.2)	0.524 (0.785, 2.759)	4.6 (1.3, 15.1)	1.464 (0.237, 4.800)
	Ph.D.	34.3 (28.1, 41.1)	4.927 (3.641, 6.611)	40.3 (32.5, 48.6)	3.654 (2.567,5.167)	21.0 (12.7, 32.6)	8.155 (4.179, 14.855)

Abbreviations: OR, odds ratio; BMI, body mass index; CI: confidence interval; Ref.: reference.

**Table 3:** Multivariate analysis of factors associated with excessive screen time.

Variable	All Participants	Males	Females
	OR (95% CI)	OR (95% CI)	OR (95% CI)
Male (vs. Female)	4.545 (3.953, 5.263)	-	-
Age (17–20 vs. 14–16)	0.885 (0.819, 0.955)	0.880 (0.807, 0.960)	-
Grade		-	-
Second year (vs. First year)	0.593 (0.506, 0.694)	0.577 (0.482, 0.688)	0.625 (0.449, 0.861)
Third year (vs. First year)	0.568 (0.484, 0.667)	0.602 (0.502, 0.721)	0.427 (0.313, 0.578)
Fourth year (vs. First year)	0.934 (0.531, 1.575)	1.011 (0.542, 1.801)	0.527 (0.105, 1.729)
Residence on campus (yes vs. no)	1.716 (1.469, 2.010)	1.814 (1.536, 2.152)	1.640 (1.181, 2.311)
Physical disease (yes vs. no)	1.569 (1.276, 1.918)	1.461 (1.151, 1.840)	1.884 (1.226, 2.805)
Chronic constipation (yes vs. no)	1.512 (1.268, 1.796)	1.631 (1.332, 1.987)	-
Smoking (yes vs. no)	1.474 (1.180, 1.836)	1.423 (1.122, 1.798)	2.328 (1.261, 4.165)
Alcohol consumption (yes vs. no)	1.789 (1.530, 2.088)	1.658 (1.400, 1.960)	2.764 (1.850, 4.027)
Sexual activity (ever active vs. never active)	1.743 (1.400, 2.158)	1.768 (1.396, 2.228)	-
Inhabitation			
Rural (vs. urban)	1.069 (0.930, 1.229)	-	0.995 (0.733, 1.357)
Rural–urban continuum (vs. urban)	1.316 (1.003, 1.710)	-	2.361 (1.430, 3.768)
Paternal education			
Junior college (vs. High school and below)	0.734 (0.582, 0.918)	0.820 (0.636, 1.048)	0.450 (0.260, 0.749)
Bachelor (vs. High school and below)	1.219 (0.943, 1.567)	1.250 (0.938, 1.656)	1.023 (0.591, 1.734)
Master (vs. High school and below)	0.808 (0.388, 1.562)	0.620 (0.261, 1.337)	1.492 (0.320, 5.001)
Ph.D. (vs. High school and below)	1.574 (0.780, 3.110)	1.247 (0.572, 2.642)	5.857 (0.974, 29.922)
Maternal education			
Junior college (vs. High school and below)	0.815 (0.638, 1.034)	0.661 (0.500, 0.867)	1.674 (1.024, 2.673)
Bachelor (vs. High school and below)	0.673 (0.507, 0.887)	0.521 (0.375, 0.716)	1.445 (0.817, 2.509)
Master (vs. High school and below)	1.390 (0.663, 2.749)	1.973 (0.880, 4.203)	0.231 (0.011, 1.517)
Ph.D. (vs. High school and below)	2.438 (1.221, 4.902)	2.628 (1.234, 5.671)	1.392 (0.246, 8.412)

OR, odds ratio; CI: confidence interval.

**Table 4:** The positive rate of mental health problems and urinary incontinence in different ST groups and gender.

Variable	Total			Males			Females		
	Excessive ST (*n* = 1410)	Low ST ^§^ (*n* = 13,645)	*p* Value *	Excessive ST (*n* = 1148)	Low ST (*n* = 6366)	*p* Value	Excessive ST (*n* = 262)	Low ST (*n* = 7279)	*p* Value
**Mental Health problems**	820 (58.2)	5467 (40.1)	<0.001	650 (56.6)	2605 (40.9)	<0.001	170 (64.9)	2862 (39.3)	<0.001
Academic stress	1052 (74.6)	7820 (57.3)	<0.001	847 (73.8)	3588 (56.4)	<0.001	205 (78.2)	4232 (58.1)	<0.001
Emotional disturbance	994 (70.5)	7361 (53.9)	<0.001	791 (68.9)	3472 (54.5)	<0.001	203 (77.5)	3889 (53.4)	<0.001
Obsessive–compulsive tendencies	904 (64.1)	7108 (52.1)	<0.001	730 (63.6)	3303 (51.9)	<0.001	174 (66.4)	3805 (52.3)	<0.001
Anxiety	892 (63.3)	7051 (51.7)	<0.001	707 (61.6)	3134 (49.2)	<0.001	185 (70.6)	3917 (53.8)	<0.001
Maladaptation	933 (66.3)	6225 (45.6)	<0.001	754 (65.7)	3039 (47.7)	<0.001	179 (68.3)	3186 (43.8)	<0.001
Interpersonal sensitivity	890 (63.1)	6213 (45.5)	<0.001	714 (62.6)	2908 (45.7)	<0.001	176 (67.2)	3305 (45.4)	<0.001
Paranoid ideation	800 (56.7)	5439 (39.9)	<0.001	643 (56.0)	2669 (41.9)	<0.001	157 (59.9)	2770 (38.1)	<0.001
Depression	748 (53.0)	5403 (39.6)	<0.001	568 (49.5)	2306 (36.2)	<0.001	180 (68.7)	3097 (42.5)	<0.001
Hostility	629 (44.6)	4479 (32.8)	<0.001	483 (42.1)	2135 (33.5)	<0.001	146 (55.7)	2344 (32.2)	<0.001
Psychological imbalance	577 (40.9)	4002 (29.3)	<0.001	471 (41.0)	2091 (32.8)	<0.001	106 (40.5)	1911 (26.3)	<0.001
**UI**	129 (9.1)	866 (6.3)	<0.001	102 (8.9)	352 (5.5)	<0.001	27 (10.3)	514 (7.1)	0.046
Stress UI	16 (1.1)	238 (1.7)	0.091	9 (0.8)	47 (0.7)	0.868	7 (2.7)	191 (2.6)	0.962
Urgency UI	100 (7.1)	560 (4.1)	<0.001	83 (7.2)	269 (4.2)	<0.001	17 (6.5)	291 (4.0)	0.045
Mixed UI	13 (0.9)	68 (0.5)	0.060	10 (0.9)	36 (0.6)	0.310	3 (1.1)	32 (0.4)	0.235

Abbreviations: ST, screen time; UI, urinary incontinence. ^§^ screen time ≤ 2 h/day. * *p* value is calculated from proportion test.

## Data Availability

Not applicable.
